# The C-terminal amyloidogenic peptide contributes to self-assembly of *Avibirnavirus* viral protease

**DOI:** 10.1038/srep14794

**Published:** 2015-10-06

**Authors:** Xiaojuan Zheng, Lu Jia, Boli Hu, Yanting Sun, Yina Zhang, Xiangxiang Gao, Tingjuan Deng, Shengjun Bao, Li Xu, Jiyong Zhou

**Affiliations:** 1 Key Laboratory of Animal Virology of Ministry of Agriculture, Zhejiang University, Hangzhou 310058, PR China; 2 State Key Laboratory and Collaborative Innovation Center for Diagnosis and Treatment of Infectious Diseases, First Affiliated Hospital, Zhejiang University, Hangzhou 310003, PR China; 3 College of Veterinary Medicine, Nanjing Agricultural University, Nanjing 210095, PR China

## Abstract

Unlike other viral protease, *Avibirnavirus* infectious bursal disease virus (IBDV)-encoded viral protease VP4 forms unusual intracellular tubule-like structures during viral infection. However, the formation mechanism and potential biological functions of intracellular VP4 tubules remain largely elusive. Here, we show that VP4 can assemble into tubules in diverse IBDV-infected cells. Dynamic analysis show that VP4 initiates the assembly at early stage of IBDV infection, and gradually assembles into larger size of fibrils within the cytoplasm and nucleus. Intracellular assembly of VP4 doesn’t involve the host cytoskeleton, other IBDV-encoded viral proteins or vital subcellular organelles. Interestingly, the last C-terminal hydrophobic and amyloidogenic stretch ^238^YHLAMA^243^ with two “aggregation-prone” alanine residues was found to be essential for its intracellular self-assembly. The assembled VP4 fibrils show significantly low solubility, subsequently, the deposition of highly assembled VP4 structures ultimately deformed the host cytoskeleton and nucleus, which was potentially associated with IBDV lytic infection. Importantly, the assembly of VP4 significantly reduced the cytotoxicity of protease activity in host cells which potentially prevent the premature cell death and facilitate viral replication. This study provides novel insights into the formation mechanism and biological functions of the *Avibirnavirus* protease-related fibrils.

The protein aggregation plays critically pathogenic roles in a wide range of increasingly prevalent diseases, including amyloid-beta peptide and hyperphosphorylation of Tau protein in Alzheimer’s disease, alpha-synuclein protein in Parkinson’s disease, islet amyloid polypeptide in type-II diabetes, prion protein in transmissible spongiform encephalopathies and mutant P53 protein in some cancers *et al.*^1^,^2^. Importantly, increasing evidences indicate the association of protein aggregation with infectious disease. The most pertinent example is the crucial role of amyloid in human immunodeficiency virus (HIV) infection and transmission. A semen amyloid named “semen-derived enhancer of virus infection” greatly enhances HIV transmission by self-assembly of prostatic acid phosphatase^3^,^4^, and peptides derived from HIV-1 gp120 co-receptor binding domain form amyloid fibrils and enhance HIV-1 infection^5^. Besides, β-amyloid fibrils derived from Alzheimer’s disease can enhance viral infection by facilitating viral attachment and entry for various enveloped viruses^6^. Interestingly, amyloid-related tau protein seems to facilitate the spread of Alzheimer’s disease like an infection from neuron to neuron^7^,^8^. Here we described a novel evidence of the intracellular assembly of fibrils for viral protease encoded by an *Avibirnavirus*.

*Avibirnavirus* or infectious bursal disease virus (IBDV), a representative member of *Birnaviridae* family, is highly contagious pathogen which can damage the precursors of antibody-producing B lymphocytes and cause severe immunosuppression and mortality in young chickens^9^. IBDV-encoded VP4 has been identified as a serine protease which utilizes a unique serine/lysine catalytic dyad mechanism to process the viral polyprotein precursor (NH2-pVP2-VP4-VP3-COOH) and capsid precursor pVP2, and play critical roles in virion assembly and maturation^10^,^11^. Recently, we further found that VP4 is a phosphoprotein with phosphorylated sites ^26^S, ^99^Y and ^162^T during IBDV infection, and the phosphorylation of ^99^Y and ^162^T partially contributes to the cleavage of intermediate precursor VP4-VP3 polyprotein^12^. VP4 can also interact with host glucocorticoid-induced leucine zipper and suppress expression of type I interferon in IBDV-infected HEK293T cells to facilitate IBDV replication^13^. During IBDV assembly, viral ribonucleoprotein VP3 and capsid precursor pVP2, together with viral dsRNA and viral polymerase VP1, are effectively encapsidated into virus-like particles^14^,^15^,^16^. However, VP4 protein forms 24–26 nm type-II tubules in the cytoplasm and nucleus of IBDV-infected cells which are different from type-I virion-related tubules and fails to be eventually encapsidated into infectious IBDV virions^17^. Recent studies using VP4 protein expressed in *Escherichia coli (E. coli)* showed that VP4 possess the capacity to self-assemble into the tubular structures^18^, and *in vitro* assays revealed that VP4 tubules had much lower endopeptidase and protease activity than monomeric and dimeric VP4^19^. Our recent study showed that ^26^S, ^99^Y and ^162^T phosphorylation of VP4 protein was nonessential for VP4 assembly^12^. Thus, both formation mechanism and potential functions of intracellular VP4-related tubules during IBDV infection largely remain unclear. Here we systematically characterized the dynamic assembly of intracellular VP4 tubules. Importantly, VP4 were convinced to possess an amyloidogenic peptide at the C-terminus which is critical for VP4 assembly. The assembled VP4 tubules show significantly low solubility, and the accumulating deposition of VP4 tubules eventually destroys host cytoskeleton and nucleus during late stage of IBDV infection. VP4 assembly initiated at early stage of IBDV infection greatly reduces the cytotoxic effects of protease on host cells, which potentially prevents premature cell death and facilitates viral replication.

## Results

### Intracellular assembly of viral protease VP4 is independent of cell types

**Figure 1:**
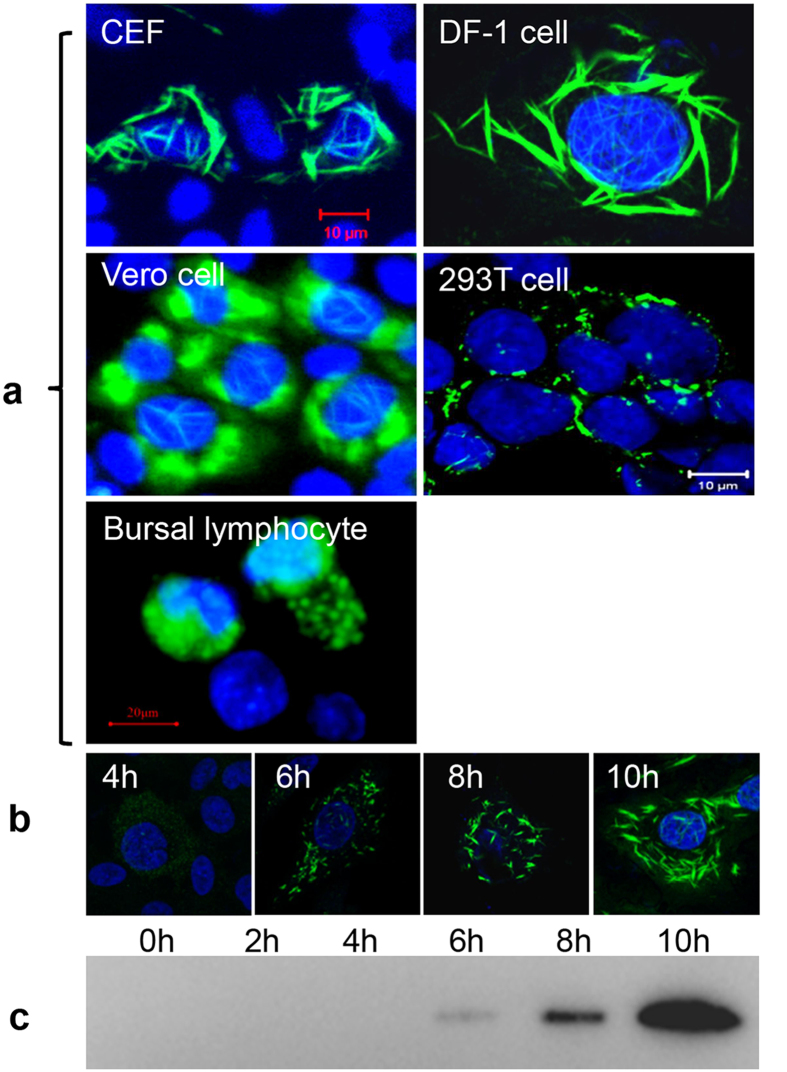
Expression and subcellular dynamics of VP4 in IBDV-infected cells. (**a**) Subcellular expression of VP4 in IBDV-infected CEF (24 h), DF-1 cells (24 h), Vero cells (72 h), 293T cells (24 h) and intrabursal lymphocytes (72 h). (**b**) Assembly dynamics of VP4 in IBDV-infected DF-1 cells was examined at 4, 6, 8 and 10 h after infection (MOI = 1) by IFA using anti-VP4 mAb, and nuclei were stained with DAPI. (**c**) Expression dynamics of VP4 in IBDV-infected DF-1 cells (MOI = 1) was examined at 0, 2, 4, 6, 8 and 10 h after infection by Western blotting using anti-VP4 mAb.

VP4 was previously reported to form tubule-like structures within IBDV-infected chicken embryo fibroblast cells (CEFs)^17^, but it’s unclear if the intracellular formation of tubular VP4 is specific for certain type of cells. We characterized VP4 expression in various IBDV-infected cells, including primary CEFs, DF-1 cells, Vero cells, HEK293T cells (*in vitro*) and intrabursal lymphocytes in chicken (*in vivo*) by immunofluorescence assay (IFA) using anti-VP4 monoclonal antibody (mAb). Confocal microcopy revealed that VP4 assembles within the cytoplasm and nucleus of various IBDV-infected cells (Fig. 1a). Specifically, intranuclear VP4 assembled into varying sizes of fibrils among IBDV-infected different cells, cytoplasmic VP4 in IBDV-infected CEF, DF-1 cells and HEK293T cells mainly formed needle-like fibrils, while cytoplasmic VP4 in the IBDV-infected Vero cells and intrabursal lymphoid cells formed large mass-like aggregates. These data demonstrated that intracellular VP4 assembly is an intrinsic feature of IBDV infection which is independent of cell types.

### Intracellular assembly of VP4 is initiated at an early stage of IBDV infection

Having demonstrated the structural similarities of VP4 among various IBDV-infected cells, we subsequently used the DF-1 cell line as a model system to analyze the detailed dynamics of VP4 structure formation. DF-1 cells were infected with IBDV at a multiplicity of infection (MOI) of 1 and intracellular expression and distribution of VP4 were detected by IFA and confocal microscope at 2 to 24 hours post infection (hpi). Figure 1b showed the dynamics of VP4 assembly within the cytoplasm and nucleus of infected cells. Specifically, VP4 seeded as small granules at 2 h and 4 h, gradually assembled from short rod-shaped structures (at 6 h) into needle-like or filamentous structures (at 8 h), and the structures become larger from 10 h onwards until 24 h. Consistent with the IFA results, Western blotting revealed that 28-kDa VP4 protein cleaved from the polyprotein precursor was detectable as early as ~6 hpi; expression levels of VP4 protein increased at 8 h and became dramatically higher at 10 h (Fig. 1c). Coincidence of visible VP4 assembly and detectable VP4 expression suggested that VP4 assembly is initiated at early stage of IBDV replication, accompanied by the proteolytic cleavage of polyprotein precursor.

### Assembly of intracellular VP4 is independent of other viral proteins or key subcellular organelles

**Figure 2:**
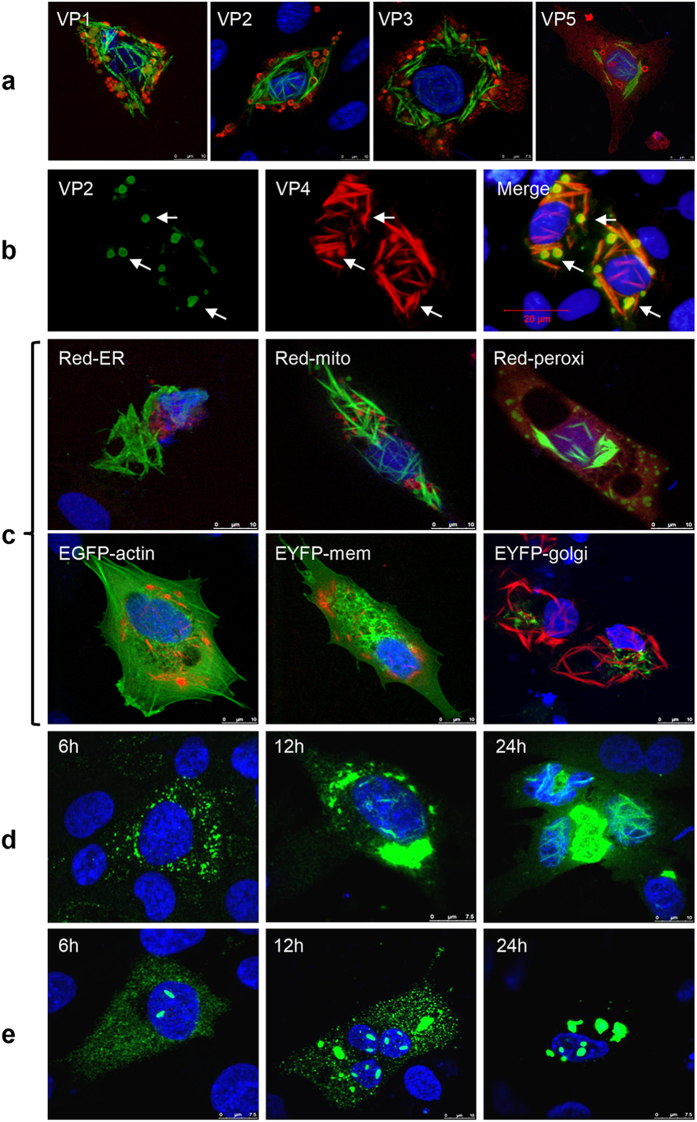
Intracellular assembly of VP4 does not involve other IBDV-encoded proteins or subcellular organelles. (**a**) No co-localization of VP4 and IBDV-encoded VP1, VP2 VP3 or VP5 was detected in IBDV-infected cells at 24 hpi. VP1, VP2, VP3 and VP5 in IBDV-infected DF-1 cells were detected with the corresponding mouse mAbs followed by TRITC-conjugated anti-mouse IgG (Red). VP4 was detected by rabbit anti-VP4 antibody followed by FITC-conjugated anti-rabbit IgG (Green). (**b**) Dot-like VP4 signal partially (white arrow) co-localize with VP2 at 24 hpi in some of IBDV-infected cells. VP2 was detected with mouse anti-VP2 mAb followed by FITC-conjugated anti-mouse IgG (Green). VP4 was detected by rabbit anti-VP4 antibody followed by TRITC-conjugated anti-rabbit IgG (Red). (**c**) Subcellular relationship of VP4 with subcellular organelles in IBDV-infected cells (MOI = 1) which were pre-transfected with living colors subcellular localization vectors pDsRed2-ER, pDsRed2-Mito, pDsRed2-Peroxi, pEGFP-actin, pEYFP-Golgi or pEYFP-Mem. Cells were fixed and stained with mouse anti-VP4 mAb 24 hpi and FITC/TRITC-conjugated goat anti-mouse IgG. (**d**) Expression kinetics of VP4 in pCI-wtVP4-transfected DF-1 cells. VP4 was stained at 6, 12 and 24 hpi with anti-VP4 mAb followed by FITC-conjugated anti-mouse IgG. (**e**) Expression kinetics of EGFP-VP4 protein in pEGFP-wtVP4-transfected DF-1 cells. Rod-shaped and mass-like structures of EGFP-VP4 protein were present in the cytoplasm and nucleus at 6, 12 and 24 h after transfection. Nuclei were stained with DAPI.

His-tagged VP4 proteins expressed in *E. coli* can self-assemble into tubule-like structures^18^ which are very similar to tubules found in IBDV-infected cells^17^. However, it is unclear if other viral proteins or intracellular factors are involved in the VP4 assembly during IBDV replication. Firstly, we examined the subcellular relationship of tubule-like VP4 structures with other IBDV-encoded viral proteins in infected DF-1 cells by dual-staining IFAs. Co-localization analysis showed that the needle-like VP4 structures did not overlap with viral proteins VP1, VP2, VP3 or VP5 (Fig. 2a), only dot-like VP4 structures co-localize with the VP2 at 24 hpi (Fig. 2b). To further analyze the relationship of VP4 structures with subcellular organelles, DF-1 cells were infected with IBDV after individually transfected with the living colors subcellular localization vectors pDsRed2-ER, pDsRed2-Mito, pEGFP-actin, pDsRed2-Peroxi, pEYFP-Golgi or pEYFP-Mem. Confocal microscopy of VP4-stained cells revealed no co-localization of VP4 with host endoplasmic reticulum (ER), mitochondria (Mito), actin, peroxisomes (Peroxi), Golgi apparatus (Golgi) or membrane (Mem) (Fig. 2c), indicating that VP4 assembly is not directly associated with other viral proteins or host organelles. To further investigate whether intracellular VP4 protein alone can form these unique structures, VP4 expression in DF-1 cells transfected with pEGFP-VP4 or pCI-VP4 was detected at 6, 12 and 24 hours post transfection (hpt). Confocal microscopy showed that wild-type VP4 (wtVP4) proteins with or without an enhanced green fluorescent protein (EGFP) tag at the N terminus can assemble within the cytoplasm and nucleus of transfected cells. Specially, VP4 assembly was initiated before 6 h, both wtVP4 (Fig. 2d) and EGFP-wtVP4 (Fig. 2e) gradually assembled from small granules into filamentous or mass-like structures. Under a living-cell confocal microscope, the intracellular EGFP-wtVP4 fusion protein was dynamically observed to gradually aggregate within the cytoplasm and nucleus, and coalesce into larger mass-like structures (Supplementary Video S1). The dynamic expression of EGFP-VP4 or wtVP4 in transfected cells (Fig. 2d,e, Supplementary Video S1) was similar to those in IBDV-infected cells (Fig. 1b). These results indicate that intracellular VP4 can self-assemble into unique structures within the cytoplasm and nucleus which is independent of IBDV-encoded viral proteins and host organelles.

### The last C-terminal hydrophobic residues are necessary for intracellular VP4 self-assembly

**Figure 3:**
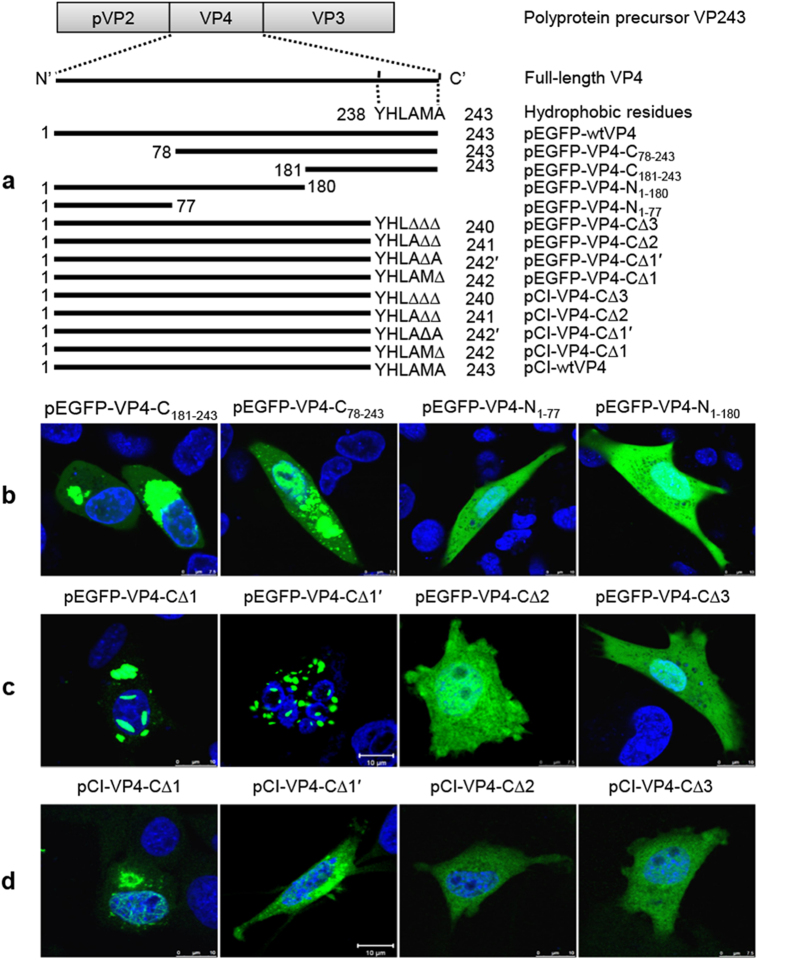
Functional mapping of the critical residues for VP4 assembly. (**a**) Schematic diagram of recombinant plasmids with full-length VP4 (wtVP4), truncated VP4 mutants and serial deletion mutants at the C-terminus of IBDV VP4. The amino acids of the hydrophobic stretch at the C-terminus of VP4 are shown. Full-length and mutant VP4 sequences were amplified and subcloned into pEGFP-C2 and pCI-neo vectors to generate the indicated recombinant plasmids. (**b**) Subcellular expression of truncated VP4 mutants in DF-1 cells at 24 h after transfection. The larger mass-like VP4 protein was present in pEGFP-VP4-C_78-243_- and pEGFP-VP4-C_181-243_-transfected cells, but not in pEGFP-VP4-N_1-77_- and pEGFP-VP4-N_1-180_-transfected cells. (**c**) EGPF-VP4 expression in DF-1 cells 24 h after transfection with C-terminal amino acid deletion mutants. The rod-shaped and mass-like VP4 structures appeared in pEGFP-VP4-C∆1- and pEGFP-VP4-C∆1′-transfected DF-1 cells, but not in pEGFP-VP4-C∆2- and pEGFP-VP4-C∆3-transfected DF-1 cells. (**d**) VP4 expression in DF-1 cells 24 h after transfection with the mutant pCI-VP4-C∆1, pCI-VP4-C∆1′, pCI-VP4-C∆2 and pCI-VP4-C∆3. The filamentous, rod-shaped and mass-like structures of VP4 appeared in pCI-VP4-C∆1- and pCI-VP4-C∆1′-transfected DF-1cells, but not in cells transfected with pCI-VP4-C∆2 or pCI-VP4-C∆3. All cells were labeled with mouse anti-VP4 mAb. Nuclei were stained with DAPI.

To identify the critical regions for intracellular VP4 self-assembly, four VP4 truncated mutants (pEGFP-VP4-N_1–77_, pEGFP-VP4-N_1–180_, pEGFP-VP4-C_181–243_ and pEGFP-VP4-C_78–243_) were constructed (Fig. 3a) and transfected into DF-1 cells. EGFP-VP4-N_1–77_ and EGFP-VP4-N_1–180_ were diffusely distributed within the cytoplasm and nucleus, while the majority of EGFP-VP4-C_181–243_ and EGFP-VP4-C_78–243_ proteins aggregated into larger mass-like structures within the cytoplasm and nucleus (Fig. 3b), indicating that the C-terminus of VP4 contributes to its intracellular assembly. Our results using intracellular system were consistent with recent results using *E. Coli*-expressed VP4 showing that C-terminal 28mer of VP4 is important for its extracellular self-assembly^19^. Intriguingly, sequence analysis revealed that the last C-terminal residues ^238^YHLAMA^243^ of VP4 form a highly hydrophobic stretch, and sequence alignment indicated that the C-terminal hydrophobic residues ^238^Y and ^240^LAMA^243^ are completely conserved among all isolated IBDV strains, and only ^239^H are mutated into ^239^D in very-virulent strains. All these information suggested that the C-terminal hydrophobicity potentially plays key role in the VP4 assembly. To further confirm this hypothesis, a series of C-terminal deletion mutants were subcloned into both pEGFP-C2 and pCI-neo expression systems, respectively, the resulting mutants (pEGFP-VP4-C∆1, ∆1′, ∆2, ∆3 and pCI-VP4-C∆1, ∆1′, ∆2, ∆3) and transfected into DF-1 cells (Fig. 3a). In both expression systems (Fig. 3c,d), deletion of the single C-terminal residue ^242^M (∆1′) or ^243^A (∆1) did not disturb VP4 assembly, but C-terminal deletion of two (^242^MA^243^), three (^241^AMA^243^) or more than three residues caused failure of VP4 assembly. These results suggest that the last hydrophobic residues at the C-terminus are critical for VP4 assembly.

### The last C-terminal hydrophobic stretch of VP4 is an amyloidogenic peptide

**Figure 4:**
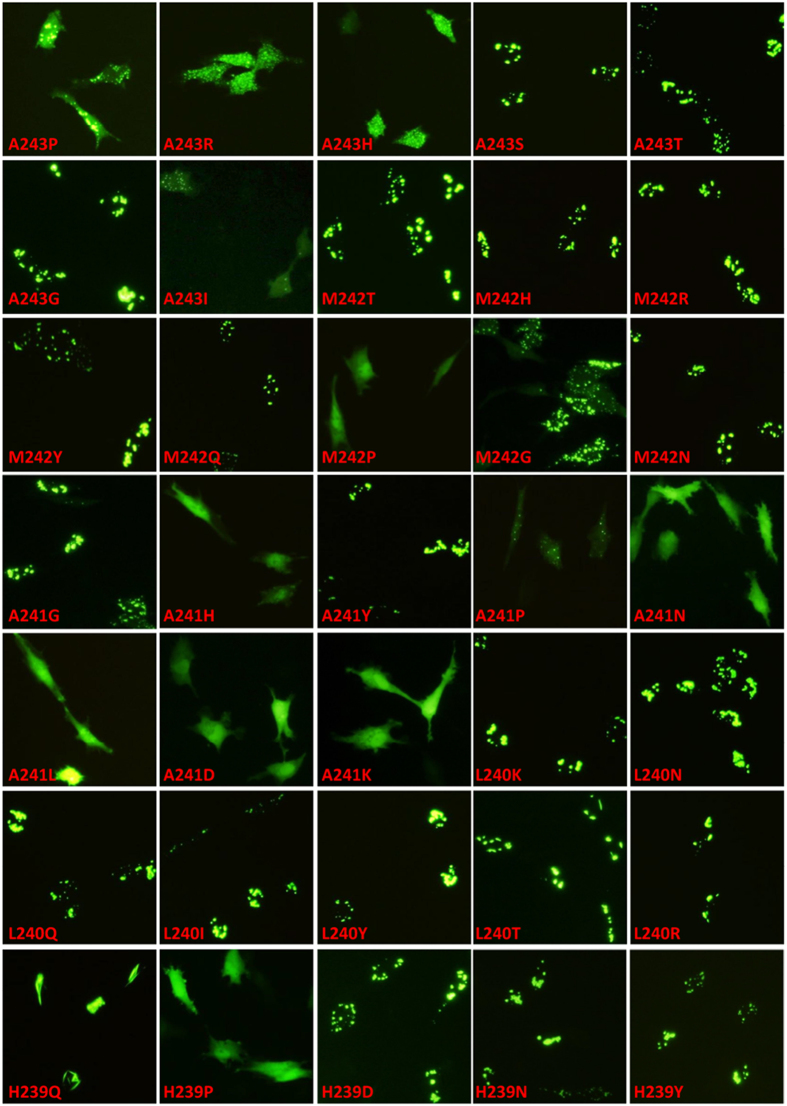
Mutational effects of C-terminal amyloidogenic peptide on VP4 assembly. Each residue of the last C-terminal stretch ^239^HLAMA^243^ is randomly mutated into NNK degenerate codon, the mutation effects of individual mutants on self-assembly were examined at 12 h after transfecting DF-1 cells with indicated pEGFP plasmids.

We attempted to predict the possibility of amyloidogenic peptides for VP4 protein using the online prediction tool which utilizes five independently published methods^20^. Consensus prediction of VP4 protein primary sequence revealed 8 potentially amyloidogenic peptides (aa 7–13, 36–40, 45–54, 67–71, 113–117, 143–151, 206–210 and 238–242). Importantly, C-terminal residues ^238^YHLAM^242^ of VP4 are highly predicted as a top hit for amyloid propensity based on two independent methods, including the average packing density value^21^ and conformational energy values^22^, and A^243^ is predicted as hit by conformational energy values. The consensus prediction of amyloidogenic regions ^238^YHLAMA^243^ is also consistent with the results of deletion mutation (Fig. 3d). To further define the property of C-terminal amino acids for VP4 assembly, each of C-terminal residues ^239^HLAMA^243^ was randomly substituted with degenerate codon NNK (N = A/C/T/G, K = G/T) and respectively subcloned into pEGFP-C2 vector. The resulting clones of VP4 random mutants were individually transfected into DF-1 cells to investigate how the mutations affect VP4 self-assembly (Fig. 4). Aromatic, hydrophobic and electrostatic interaction were known to play essential roles in the formation of amyloid fibrils^23^,^24^. Our mutagenic analysis extensively confirmed that substitutions of the C-terminal residues with aromatic residues (M242Y, A241Y, L240Y and H239Y) or electrostatic residues (M242N, L240N, H239N, M242Q, L240Q and H239Q) maintain the capability of fibril assembly (Fig. 4). Proline (P) residue was generally known to have the helical-breaking and β-sheet-disrupting effects which will destabilize amyloid fibrils^25^,^26^. As expected, substitutions of H^239^, A^241^, M^242^ or A^243^ with proline residue result in destruction of VP4 self-assembly (Fig. 4). Small amino acids, including alanine and glycine, were reported to be important for self-assembly, and thus commonly used as aggregation-prone system to study self-assembly^27^. As expected, substitution of ^240^LAMA^243^ with glycine (L240G, A241G, M242G and A243G) maintained the VP4 self-assembly (Fig. 4). Additionally, the substitutions of A^241^ and A^243^ with charged amino acids (A243H, A243R, A241H, A241K and A241D), some hydrophobic residues (A243I and A241L), or electrostatic residue (A241N) mostly abolish the VP4 assembly, while M^242^, L^240^ and H^239^ tolerate various substitutions except proline (Fig. 4). Although H239D mainly exists in very virulent IBDV strains, the VP4-H239D aggregates like wt-VP4, thus suggesting position 239 of VP4 may be associated with virus virulence via aggregation-independent mechanism (Fig. 4). In summary, these results indicate that C-terminal stretch of VP4 has the amyloidogenic properties which are dominated by the two aggregation-prone alanine residues, ^241^A and ^243^A.

### Assembled VP4 structures show low solubility

**Figure 5:**
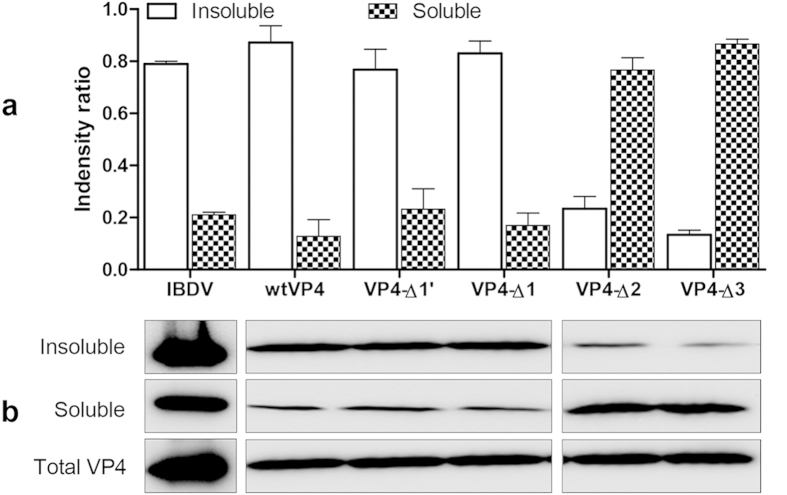
Assembly greatly decreases the solubility of VP4 protein. Plasmids pEGFP-wtVP4, pEGFP-VP4-C∆1, pEGFP-VP4-C∆1′, pEGFP-VP4-C∆2 and pEGFP-VP4-C∆3 were transfected into DF-1 cells, and IBDV-infected cells were used as a control. Cells were lysed with TX-100-containing lysis buffer at 24 h after transfection or infection. The TX-100-insoluble and TX-100-soluble fractions were analyzed by 12% SDS-PAGE and Western blotting using anti-VP4 mAb. (**a**) Volume quantification of VP4 bands was detected using Quantity One software (N = 2). (**b**) Western blots of bands corresponding to VP4. Soluble VP4 protein was increased in DF-1 cells transfected with pEGFP-VP4-C∆2 and EGFP-VP4-C∆3.

**Figure 6:**
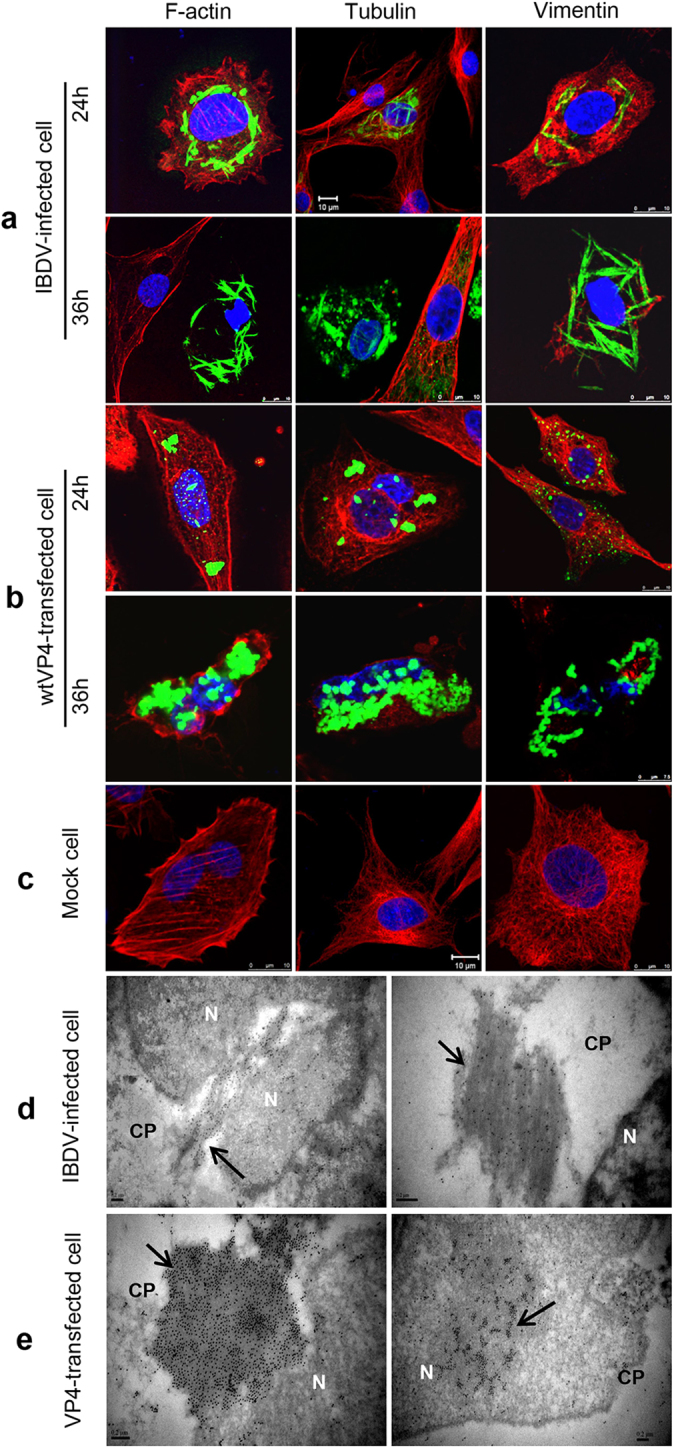
Assembled VP4 structures mechanically alter cytoskeleton and nucleus. IBDV-infected DF-1 cells (**a**), pEGFP-wtVP4-transfected DF-1 cells (**b**) and mock-infected DF-1 cells (**c**) were fixed for confocal analysis at 24 h and 36 h following infection or transfection. VP4 was detected with mouse anti-VP4 mAb or rabbit anti-VP4 polyclonal antibody followed by FITC-conjugated goat anti-mouse or anti-rabbit IgG. Host F-actin was probed with TRITC-phalloidin. Anti-β-tubulin mAb and TRITC-conjugated anti-mouse IgG were used to label host microtubules. Rabbit anti-vimentin mAb and TRITC-conjugated anti-rabbit IgG were used to stain intermediate filaments. Nuclear DNA was stained with DAPI. Scale bar is 7.5 μm for microtubules at 24 h and F-actin and vimentin at 36 h; scale bars are 10 μm for other panels. Ultrastructure of assembled VP4 in IBDV-infected DF-1 cells (**d**) and pEGFP-wtVP4-transfected DF-1 cells (**e**). The ultrathin section was stained with mouse anti-VP4 mAb followed by goat anti-mouse immunogold-labeled secondary antibody. Black arrowheads indicate bundles of VP4 tubules, N is nucleus and CP is cytoplasm.

Morphologically, the assembled VP4 structures are apparently similar to amyloid fibrils which are thought to be insoluble and resistant to degradation^28^. As VP4 was largely found to assemble within IBDV-infected cells, and maintained in the cell debris after lytic IBDV infection around 36 hpi (Fig. 6a,b), it is possibly to speculate that the assembly of VP4 affects its intracellular solubility. To test this hypothesis, DF-1 cells infected with IBDV or transfected with plasmids pCI-wtVP4, pCI-VP4-C∆3, pCI-VP4-C∆2, pCI-VP4-C∆1 and pCI-VP4-C∆1′ were lysed with RAPI lysis buffer containing 1% Triton X-100 (TX-100). VP4 protein in the TX-100-insoluble and soluble fractions were then detected by Western blotting using anti-VP4 mAb. As shown in Fig. 5, assembled VP4 proteins in IBDV-infected cells or wtVP4, VP4-C∆1 and VP4-C∆1′ in transfected cells were mainly (~80%) detected in the TX-100-insoluble fractions. In contrast, diffusely distributed VP4-C∆2 and VP4-C∆3 in transfected cells were rarely (~20%) detected in the TX-100-insoluble fraction. These results demonstrate that assembly of VP4 greatly reduced the solubility, and assembled VP4 tubules mainly exist as an insoluble component within the cells.

### Highly assembled VP4 structures mechanically deform the host cytoskeleton and nucleus at late stage

It is unclear about the exact molecular mechanism inducing cell lysis at the late stage of IBDV infection. The formation of large amount of insoluble VP4 fibrils within IBDV-infected cells ignites the hypothesis that VP4 aggregates may play roles in lytic infection. As VP4 protein mainly existed in the TX-100-insoluble pellets (Fig. 5) which are commonly considered as cytoskeletal fractions^29^,^30^, we tried to elucidate the potential relationship between assembled VP4 and the host cytoskeleton. We performed dual-staining IFA using the probe fluorescein isothiocyanate (FITC)-phalloidin for F-actin, anti-beta tubulin antibodies for microtubule and anti-vimentin antibody for intermediate filament to analyze cytoskeletal structures in IBDV-infected cells and wtVP4- and VP4-CΔ2-transfected DF-1 cells (Figs 6a,c). Unexpectedly, the assembled VP4 did not overlap with cytoskeletal proteins F-actin, beta tubulin or vimentin in either IBDV-infected cells or wtVP4-transfected DF-1 cells at 24 and 36 h after infection or transfection. Notably, at 24 h post infection or transfection, the F-actin, microtubule and intermediate filament structures in IBDV-infected and wtVP4-transfected cells (Fig. 6a,b) is similar to mock cells (Fig. 6c). After 36 h, however, accompanying the intracellular deposition of greater amounts of VP4 tubules, host F-actin, microtubules and intermediate filaments were considerably deformed or broken down in both infected and transfected cells (Fig. 6a,b). Interestingly, the cytoskeleton destruction was coincident with the cytopathic effect (CPE) in IBDV-infected DF-1 cells at ~36 hpi. To examine whether the cytoskeletal changes were related only to the assembled VP4 structures, we conducted further analysis of the subcellular relationship of cytoskeletal proteins with other IBDV-encoded proteins using cells transfected with the plasmids pCI-VP1, -VP2, -VP3 and -VP5. We found no co-localization of viral proteins VP1, VP2, VP3 or VP5 with host F-actin, microtubules or intermediate filaments, and additionally there were no drastic alterations in the cytoskeleton of these transfected cells (Supplementary Fig. S1), indicating that cytoskeletal changes were mainly associated with the assembled VP4 but not the other IBDV-encoded proteins. Notably, ultrastructural analysis further revealed bundles of immunogold-labeled VP4 structures occupied the cytoplasm and nucleus of IBDV-infected cells and EGFP-VP4-transfected cells and mechanically destroyed the host nucleus (Fig. 6d). Taken together, these data suggested that assembled VP4 physically disrupts host cytoskeleton and nucleus at late stages which potentially facilitate IBDV virion release.

### VP4 with C-terminal deletion induces extensive apoptosis

**Figure 7:**
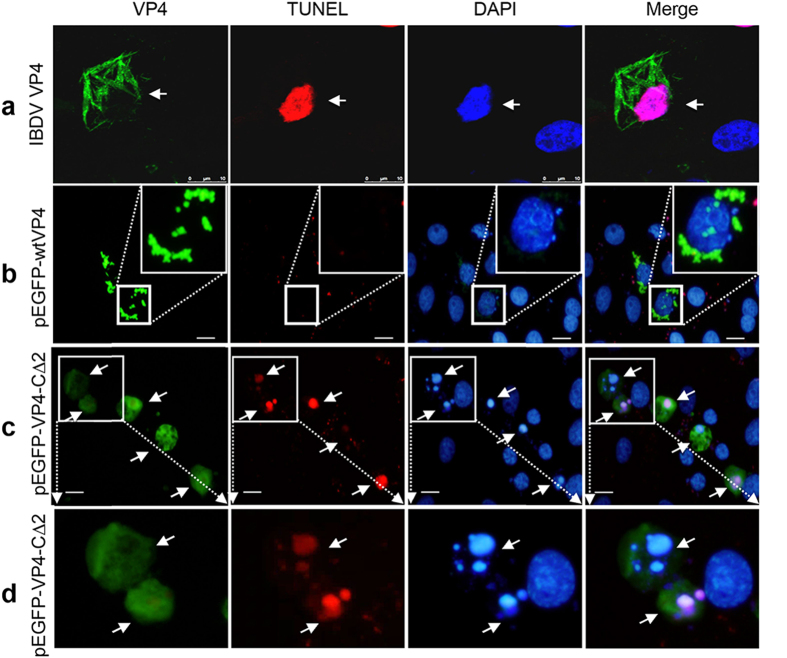
VP4-C∆2 protein without intracellular assembly induces apoptotic cell death. DF-1 cells were infected with IBDV strain NB or transfected with pEGFP-wtVP4 or pEGFP-VP4-C∆2. After 24 h, cells were fixed with 4% formaldehyde and incubated with TUNEL reaction solution to stain apoptotic cells. DAPI staining was used to demonstrate the nuclear morphology of apoptotic and normal cells. The white arrowheads indicate TUNEL-positive cells. (**a**) TUNEL assay of IBDV-infected cells. VP4 was detected using mouse anti-VP4 mAb followed by FITC-conjugated goat anti-mouse IgG. (**b**) TUNEL assay of pEGFP-wtVP4-transfected cells. The inset shows the magnification of one VP4-positive cell. (**c**) TUNEL assay of pEGFP-wtVP4-C∆2-transfected cells. The cells were TUNEL-positive and showed typical apoptotic bodies. (**d**) Magnification of TUNEL-positive cells in top left corner of panel c.

Previous report showed that IBDV-encoded serine protease VP4 did not induce apoptosis^31^. Intriguingly, VP4 with various C-terminal deletions (deletion of two or more residues) failing to assemble induced extensive cell death, including cell shrinkage and chromatin condensation (Supplementary Video S2). To further confirm the apoptotic effects, terminal deoxynucleotidyl transferase (TdT)-mediated nick end-labeling (TUNEL) assay and confocal microscopy were applied to examine apoptosis in DF-1 cells transfected with pEGFP-wtVP4 and pEGFP-VP4-C∆2, IBDV-infected cells were used as apoptosis-positive control, as IBDV infection was known to induce host apoptosis by multiple pathways^32^,^33^. As expected, IBDV-infected cells were TUNEL-positive (Fig. 7a), pEGFP-wtVP4-transfected cells showed VP4 assembly, presented a normal cellular morphology and were TUNEL-negative (Fig. 7b), whereas pEGFP-VP4-C∆2-transfected cells without VP4 assembly were TUNEL-positive, and exhibited morphological features typical of apoptosis, including cell shrinkage, chromatin condensation and apoptotic body formation (Fig. 7c,d and Supplementary Video S2). These results further confirmed that assembled VP4 fails to induce apoptosis while diffusely distributed EGFP-VP4-C∆2 was capable of eliciting apoptotic cell death.

### VP4 with C-terminal deletion maintains the cleavage activity in VP2–VP4 junctions

**Figure 8:**
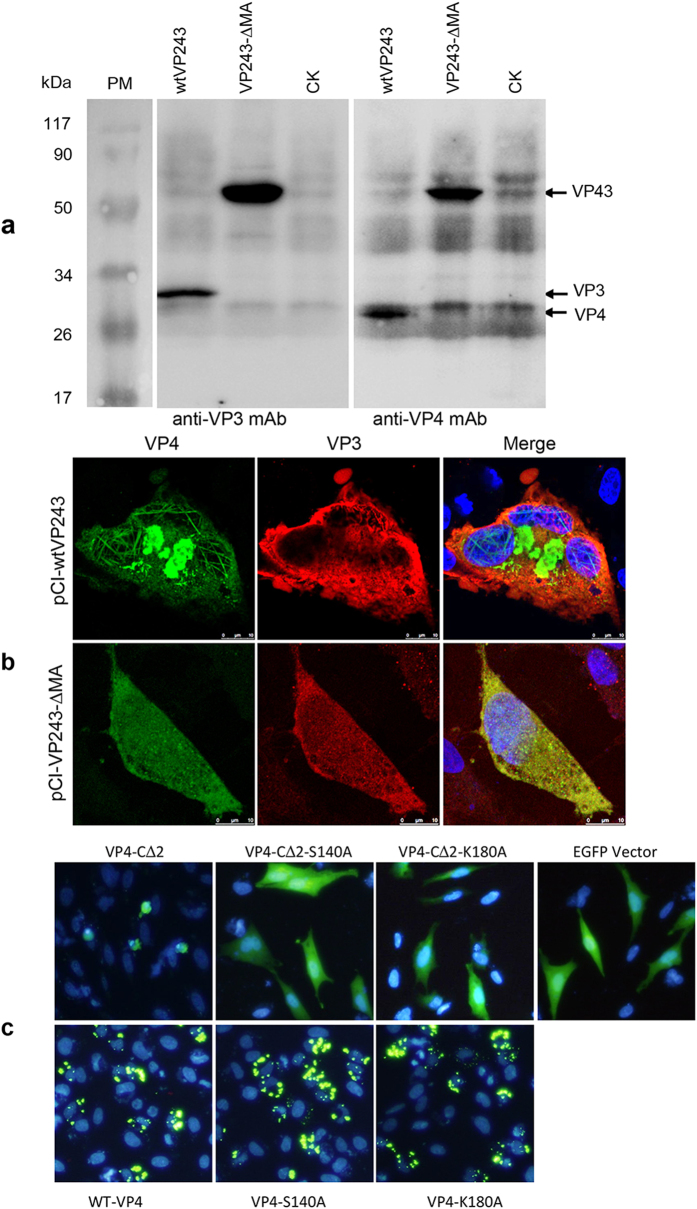
Protease activity of VP4 with C-terminal MA deletion induces cell death. (**a**) Western blotting of *in vitro* proteolytic assay of polyprotein VP243 synthesized by the TNT system. Plasmids pCI-wtVP243 and pCI-VP243-∆MA were used for *in vitro* translation to produce polyprotein VP243, and empty plasmid pCI-neo was used as negative control (CK). TNT products were subjected to Western blotting using anti-VP4 and anti-VP3 mAbs. No VP3 or VP4 protein bands were detected in the product of template pCI-VP243-∆MA. (**b**) Confocal analysis of DF-1 cells transfected with pCI-wtVP243 and pCI-VP243-∆MA. The transfected cells were fixed after 24 h, and incubated with a mixture of rabbit anti-VP3 antiserum and mouse anti-VP4 mAb followed by FITC-conjugated goat anti-mouse IgG and TRITC-conjugated goat anti-rabbit IgG. Nuclear DNA was stained with DAPI. (**c**) Substitution of protease activity site S140 or K180 of VP4 or VP4-C∆2 with alanine (A) failed to induce cell death in transfected DF-1 cells. DF-1 cells transfected with indicated mutants were directly observed under microscopy at 24 h following transfection. Empty plasmid pEGFP-C2 was used as control.

A recent study using *in vitro* assay of *E. Coli*-derived VP4 showed that VP4 tubules have significantly lower endopeptidase than monomeric or dimeric VP4^18^, which provides extracellular evidence that VP4 self-assembly potentially blocks VP4 protease activity. The proteolytic activity of proteases is important for inducing apoptotic cell death^34^, it raised our hypothesis that the apoptosis induced by intracellular VP4-C∆2 may relate to its protease activity. Firstly, we determine the proteolytic capacity of VP4-C∆2 in processing polyprotein VP243 using the TNT system. Although our anti-VP2 mAb unfortunately failed to react with VP2 protein in Western blots, protein bands with the expected sizes of VP4 (approximately 28 kDa) and VP3 (approximately 32 kDa) were detected in the product of template pCI-wtVP243 (Fig. 8a), and no more bands with larger size were detected, indicating that VP4 and VP3 were successfully and completely released from wtVP243. Conversely, approximately 60 kDa of VP4-VP3 polyprotein bands were detected by both anti-VP4 and anti-VP3 mAbs in the product of the pCI-VP243-∆MA template, but no single VP4 or VP3 bands were individually detected (Fig. 8a), indicating that VP4-VP3 junction released from VP243-∆MA precursor was not successfully cleaved into VP4 and VP3. Consistent results were obtained in cells transfected with pCI-A and pCI-A-∆MA which contain the full-length A segment or A segment with MA deletion from the C-terminus of VP4. The results further revealed that VP4 in pCI-A-transfected cells was successfully released and assembled into structures (Fig. 8b), which were similar to the VP4 structures in pCI-wtVP4-transfected cells (Fig. 2d). However, VP4 in pCI-A-∆MA-transfected cells failed to assemble into specific structures (Fig. 8b), indicating MA deletion from VP4 C-terminus affects the VP4-VP3 cleavage, which was consistent with the destruction of cleavage motif (Thr/Ala)–X–Ala↓Ala motifs^11^. Overall, these data suggested that deletion of the C-terminal residues ^242^MA^243^ of VP4 maintains the cleavage activity which can efficiently cleave VP2–VP4 junctions but not VP4–VP3 junctions with destructive cleavage motif.

### Assembly of VP4 eliminates the cytotoxicity effect of VP4 protease to prevent premature cell death

Since VP4 with C-terminal MA deletion maintain the protease activity cleaving VP2–VP4 junctions, it is reasonable to speculate that apoptosis induced by VP4-C∆2 may be triggered by its protease activity. Active sites Ser-652 and Lys-692 (corresponding position in the 243-mer VP4 is Ser-140 and Lys-180) in the serine/lysine catalytic dyad of IBDV protease are indispensable for cleavage activity^11^. To further examine if the protease activity of intracellular VP4 induce cell death, the mutants VP4-S140A, VP4-K180A, VP4-C∆2-S140A and VP4-C∆2-K180A with EGFP tag were respectively transfected to DF-1 cells, and microscope analysis (Fig. 8c) were directly applied to evaluate the cytotoxicity effects of VP4 expression on viability of DF-1 cells. Comparing with VP4-C∆2 inducing extensive apoptotic cell death, S140A and K180A mutation of VP4-C∆2 without protease activity, similar to pEGFP-C2-transfected cells, fail to induce cell death; whereas, S140A and K180A mutation of full-length VP4 showing assembled tubules but without protease activity, show similar characteristics with assembled wtVP4 tubules, failed to induce cell death. These data conversely approved that protease activity of VP4 cause cytotoxicity and thus induce the cell death; whereas assembly of VP4 potentially inactivates the protease activity. Since VP4 assembly is initiated at early stage of IBDV infection (Fig. 1b,c), the inactivation of protease activity via assembly thus prevents the premature cell death.

## Discussion

This study systematically characterized the mechanism and potential functions of intracellular assembly of the unique fibrils for *Avibirnavirus*-encoded viral protease VP4. The last C-terminal amyloidogenic stretch ^239^HLAMA^243^, especially two “aggregation-prone” alanine residues, was found to be critical for VP4 assembly. The assembly initiated at early stage of IBDV infection effectively inactivate the protease activity to eliminate the cytotoxic effects of protease on host cells, thus to prevent premature cell death; Meanwhile, the assembled VP4 fibrils are highly insoluble and the accumulating deposit of intracellular VP4 fibrils finally destroy the host cytoskeletal and nuclear structures which potentially be associated with IBDV lytic infection. This study provides new insights into the molecular mechanism and pathogenesis of IBDV replication.

Aggregations play crucial roles in the pathogenesis of multiple important neurodegenerative diseases. It is intriguing for IBDV protease to uniquely assemble into tubular structures during infection. Currently, few clues are available about the formation mechanism and potential functions for these particular structures of IBDV protease, although homotypic interaction of VP4 has been reported using the yeast two-hybrid system which suggesting the possibility of self-association between IBDV VP4 molecules^35^. Interestingly, the tubular VP4 structures in IBDV-infected target cells (Fig. 1) or VP4-transfected cells (Fig. 3) morphologically resemble amyloid-like fibrils. Multiple amyloid predictions, deletion mutants and point mutations confirmed that C-terminal stretch of IBDV VP4 forms an amyloidogenic peptide which is necessary for VP4 self-assembly (Figs 3 and 4). Solubility analysis further showed that assembled VP4 tubules are highly insoluble resembling amyloid-like structures. Additional analysis will be helpful to determine if VP4 fibrils are amyloid-like structure.

Most of viral proteases are commonly translated as part of polyproteins which will be further processed to release the viral structural proteins and viral protease. Undoubtedly, if not effectively degraded or packaged in virions, large amount of viral protease will accumulate within the infected cells and potentially induce premature cell death. Currently, the fate or degradation of viral protease during virus infection is largely elusive. Aggregation formation renders the misfolded proteins to escape the cellular quality-control mechanisms^36^ and confer resistance to degradation by the host clearance system, which is usually associated with the ubiquitin-proteasome pathway (UPP) or autophagy^37^. Our previous proteome data indeed demonstrated that UPP system was greatly impaired during IBDV infection^38^, indicating that host cells may lack the capacity to degrade the assembled VP4, however, there is no direct evidence showing the interaction between VP4 and host UPP system. Further studies on VP4-UPP interaction will be helpful to elucidate the biological functions and potential pathogenic effects of VP4 assembly during IBDV infection.

Increasing evidences have revealed that viral proteases can induce host apoptosis via various mechanisms. IBDV VP2 and VP5 have been associated with apoptosis during IBDV infection^33^,^39^,^40^, however, both previous reports^31^ and this studies revealed that IBDV-encoded serine protease VP4 fails to induce apoptosis, even though VP4 fibrils accumulated in the cells and cause cell lysis (Fig. 7b). Thus, it may hold true that apoptosis induced by IBDV infection is not dependent on intracellular VP4, but the apoptosis-related viral factors VP2 and VP5 during IBDV infection^33^,^39^,^40^. Interestingly, VP4 mutants with deletion of C-terminal ^242^MA^243^, which failed to assemble, induced extensive apoptotic cell death (Fig. 7c,d). Deletion of MA residues was known to interrupt the cleavage site (the residues ^242^MA^243^ correspond to ^754^MA^755^ in the motif ^754^MAA^756^) but maintain the cleavage activity in VP2-VP4 junction (Fig. 8) which actually elicit apoptosis (Fig. 7c,d). The recent evidence showed that the C-terminal 28 amino acid residues deleted VP4 expressed in *E.coli* is a complex of monomers and dimmers and has a higher protease activity than that of VP4 tubules^17^. Our site-directed mutagenesis on protease activity sites completely abolished the apoptotic induction (Fig. 8c), confirmed the roles of protease activity in inducing apoptosis. Taken together, our findings from apoptotic assays, cleavage analysis and mutation analysis infer that self-assembly of VP4 at an early stage of IBDV infection potentially reduces its capacity to induce premature cell death and thereby facilitates the production of IBDV progeny. The apoptotic mechanism of VP4 with C-terminal deletion and proteolytic activity remains to be further studied.

It is well known that birnaviruses replicate within the cytoplasm of host cells. The structural proteins VP1, VP2 and VP3 of IBDV were reported to locate exclusively within the cytoplasm during viral assembly^14^. In contrast, previous report^17^ and present study provided evidences that the nonstructural protein VP4 assemble into fibrils within the cytoplasm and nucleus of both IBDV-infected and VP4-transfected cells (Figs 1 and 2). However, the mechanism for nuclear entry of VP4 is still unknown. Prediction using the PSORT program in the Swiss/Prot database revealed that there is no nuclear localization consensus signal in VP4 molecules. Truncation mutations further demonstrated that deletion of either N-terminal or C-terminal fragments of IBDV VP4 did not disrupt intranuclear localization (Fig. 3b). Nuclear entry is likely to be an intrinsic feature of VP4, no matter whether or not VP4 is assembled into specific structures or other IBDV viral proteins are co-expressed (Figs 1, 2 and 3). Extensive existence of intranuclear VP4 potentially suggests an important role of VP4 in IBDV replication by interacting with host nucleus. In this study, our ultrastructural analysis revealed that abundant tubule-like VP4 structures mechanically damaged the host nucleus at late stages of IBDV infection or VP4 transfection (Fig. 6d), suggesting that intranuclear VP4 assembly is potentially associated with IBDV pathogenesis.

Lytic infection of IBDV in premature B lymphocyte was considered as the pathogenesis of IBDV-related disease^41^,^42^. However, the detailed molecular mechanism of cell lysis at the late stage of viral infection is still unclear. Nonstructural protein VP5 was reported to accumulate within the cytoplasmic membrane and alter the permeability of the plasma membrane, and potentially facilitate the release of viral progeny^43^, but there is no direct evidence for VP5 to cause the final collapse of infected cells. In this study, we demonstrated that the assembled VP4 structures extensively accumulate within IBDV-infected cells at the late stage of infection, and mechanically destroy host cytoskeletal elements, including microfilaments, microtubules and intermediate filaments (Fig. 6a–c). Collapse of host cytoskeleton typically affects cellular integrity, this will lead to cell lysis and facilitate virion egress at the end stage of infection^44^, thus the accumulating deposit of VP4 fibrils is considered to potentially facilitate virion release at late stage of IBDV infection.

Emerging of very virulent or variant IBDV strains increasingly disable the clinical IBDV vaccines. Thus, developing the effective antiviral drug is the promising alternative to control IBDV infection. Viral proteases play essential roles in the maturation of viral capsid precursor or cleavage of host factors to facilitate viral infection and replication, and thus become the ideal targets for antiviral drugs^45^,^46^. Additionally, protein aggregation increasingly becomes the novel target of structure-based rational drug design^47^,^48^. As the critical roles of viral protease during IBDV infection, VP4 is a promising target for anti-IBDV drug discovery, it is reasonable to design the anti-IBDV inhibitor targeting protease activity or amyloid-like property of VP4.

## Methods

### Virus, cells, animals, antibodies and vectors

The cell-adapted IBDV (strain NB-CEF37) with a TCID_50_ of 1.0 × 10^7.0^/0.1 ml and virulent IBDV (strain NB-Bursa6) with a 50% bursa lethal dose (BLD_50_) of 1.0 × 10^6.2^/0.1 ml were stored in our laboratory^49^. DF-1, Vero and HEK293T cell lines were purchased from ATCC and maintained in Dulbecco’s Modified Eagle’s Medium supplemented with 10% fetal bovine serum (Gibco/Invitrogen Grand Island, NY). Specific pathogen-free (SPF) chickens and embryonated eggs were purchased from Beijing Merial Vital Laboratory Animal Technology (Beijing, China). Primary CEFs were prepared from 10-day-old SPF embryonated chicken eggs and maintained in Hank’s medium supplemented with 8% newborn bovine serum. Anti-VP1, VP3, VP4 and VP5 mAbs and polyclonal antibodies were previously prepared in our laboratory^49^,^50^,^51^. Mouse anti-VP2 mAb was kindly provided by Prof. Gaiping Zhang (Henan Academy of Agricultural Sciences, China). Mammalian expression vector pCI-neo was purchased from Promega (Madison, WI). Recombinant plasmids pCI-A and pCI-B were constructed in our laboratory^50^,^52^. Eukaryotic expression vector pEGFP-C2 and living colors^TM^ subcellular localization vectors pDsRed2-ER, pDsRed2-Mito, pDsRed2-Peroxi, pEGFP-actin, pEYFP-Golgi and pEYFP-Mem were obtained from Clontech Laboratories (Palo Alto, CA). The animal study proposal was approved by the Institutional Animal Care and Use Committee (IACUC) of Zhejiang University (Permit Number: SYXK 2012-0178). All animal experimental procedures were performed in accordance with the Regulations for the Administration of Affairs Concerning Experimental Animals approved by the State Council of People’s Republic of China.

### Construction of recombinant plasmids expressing wild-type VP4 and truncated VP4

Construction of recombinant plasmids expressing wtVP4 and truncated VP4 was performed as described previously with some modifications^50^. Briefly, primers to amplify the wtVP4 and truncated VP4 were designed and synthesized (Supplementary Table S1). Truncated VP4 and wtVP4 were amplified using the plasmid pCI-A as a template. The PCR products were separately subcloned into the pEGFP-C2 vector and pCI-neo vector using the EcoRI/KpnI or NheI/EcoRI cloning sites. The resulting mutants were confirmed by Sanger sequencing.

### Viral infection, immunofluorescence assay and confocal microscopy

Primary CEF, DF-1, HEK293T and Vero cells were infected with the cell-adapted IBDV at a MOI of 1. Twenty one-day-old SPF chickens were intraocularly inoculated with virulent IBDV at a dose of 100 BLD_50_/0.1 ml. IBDV-infected and VP4-transfected cells were subjected to IFA or dual-staining IFA as previously described with slight modifications^38^. For dual-staining IFA, cells were fixed with 4% formaldehyde for 10 min and blocked with 5% non-fat milk in phosphate-buffered saline (PBS) containing 0.05% Tween 20 for 1 h. Cells were then incubated for 2 h with a mixture of rabbit and mouse antibodies specific for each protein. After washing five times, the cells were incubated for 1.5 h with a mixture of tetraethylrhodamine isothiocyanate (TRITC)-conjugated anti-mouse IgG and FITC-conjugated anti-rabbit IgG. TRITC-phalloidin (Sigma) was used to stain F-actin. Nuclei were stained with 10 μg/ml 4′,6-diamidino-2-phenylindole (DAPI) for 5 min. The subcellular expression and localization of proteins were subsequently examined under a Zeiss LSM 700 laser scanning confocal microscope (Carl Zeiss, Jena, Germany).

### Live-cell confocal microscopy

To visualize the dynamics of VP4 molecules in living cells, DF-1 cells growing on glass-bottomed dishes (MatTek, Ashland, MA) were transfected with the recombinant plasmids pEGFP-wtVP4 or pEGFP-VP4-CΔ2 using Lipofectamine 2000 (Invitrogen). At 8 h following transfection, the nuclei of living cells were stained with Hoechst 33342 (Sigma) for 20 min. Cells were then rinsed with PBS twice and supplied with fresh medium. The expression dynamics of EGFP-wtVP4 and EGFP-VP4-CΔ2 were visualized using a Zeiss LSM 700 laser scanning microscope in a humidified cell culture chamber with 5% CO_2_ at 37 °C.

### Western blotting

Cell lysates were subjected to 12% SDS-PAGE and bound to Hybond-C Extra nitrocellulose membranes (Amersham Biosciences, Piscataway, NJ) using a semi-dry blotting system (Amersham Biosciences). After blotting, membranes were blocked and incubated with mouse primary antibodies followed by horseradish-peroxidase (HRP)-conjugated anti-mouse IgG (KPL). Bands were visualized using SuperSignal^®^ West Femto Trial Kit (Thermo Scientific/Pierce, Rockford, IL Rockford, IL) as an enhanced chemiluminescence substrate for HRP and then scanned using the FluorChem M system (Cell Biosciences, Santa Clara, CA).

### Solubility of intracellular VP4

DF-1 cells were infected with IBDV or transfected with pCI-wtVP4, pCI-VP4-C∆3, pCI-VP4-C∆2, pCI-VP4-C∆1 or pCI-VP4-C∆1′, collected at 24 h by centrifuging at 8,000 × *g* for 5 min, lysed with RAPI lysis buffer containing 1% TX-100, 20 mM Tris pH 7.5 and 150 mM NaCl for 30 min on ice, and then centrifuged at 12,000 *g* for 20 min. The soluble fractions were precipitated with cold acetone for 30 min and centrifuged at 12,000 *g* for 20 min. The pellets of insoluble fractions and precipitated soluble fractions were dissolved with equal volume of 2-DE lysis buffer containing 7 M urea, 2 M thiourea, 4% CHAPS, 65 mM DTT, 0.2% Bio-Lyte and protease inhibitor cocktail^38^. Equal numbers of TX-100-untreated cells were lysed with 2-DE lysis buffer and used as control containing total VP4 protein. Equal amounts of protein were subjected to 12% SDS-PAGE and Western blotting using anti-VP4 mAb. Densitometric analysis of VP4 was performed using Quantity One software (Bio-Rad Laboratories, Philadelphia, PA).

### *In vitro* proteolytic assay

To determine proteolytic capacity of wtVP4 or VP4 mutants to cleave polyprotein VP243, the TNT system was used to synthesize the polyprotein VP243 *in vitro* using the recombinant plasmids pCI-VP243-∆MA and pCI-wtVP243 as templates. Firstly, to construct the mutant segment A containing the gene VP4 with deleted C-terminal amino acid residues ^242^MA^243^ (A-∆MA), the 5′-terminal and 3′-terminal segments were respectively amplified using the plasmid pCI-A as a template and the primer pairs A5/A-MA-3 and A-MA-5/A3 (Supplementary Table S1), and then the A-ΔMA segment was synthesized by a fusion PCR using primer pair A5/A3 and inserted into the pCI-neo vector. To further construct recombinant plasmids encoding polyprotein VP243, or VP243 with MA deletion at the C-terminal end of VP4, for *in vitro* proteolytic assays, the resulting plasmids pCI-A-∆MA and pCI-A were used as templates; segment A with the MA deletion (VP243-ΔMA) and without the deletion (wtVP243) were amplified using the primer pair TNT-AT7/TNT-A3m, re-amplified using the primer pair TNT-A5m/TNT-A3 (Supplementary Table S1), and then separately inserted into the pCI-neo vector. The resulting plasmids pCI-wtVP243 and pCI-VP243-∆MA were confirmed by sequencing and purified for *in vitro* transcription and translation to produce polyprotein wtVP243 or VP243-ΔMA using TNT® T7 Quick Coupled Transcription/Translation System (Promega), according to the manufacturer’s instructions. Western blotting was used to detect the products of the wtVP243 or VP243-ΔMA polyprotein using mouse anti-VP2, anti-VP4 and anti-VP3 mAbs. Meanwhile, the recombinant plasmids pCI-A and pCI-A-∆MA were also used for transfection, followed by dual-staining IFA using antibodies to VP3 and VP4. Nuclear DNA was stained by DAPI for 5 min, and then triple-stained cells were observed under a laser confocal microscope.

### Mutagenesis of protease activity sites and C-terminal residues of VP4

Site-directed mutagenesis of protease activity sites, S140A and K180A, were performed using two-step PCR. Briefly, two overlapping fragments with desired mutations were obtained by two independent PCR using the primer pair pEGFP-wtVP4-F/S140A-R and S140A-F/pEGFP-wtVP4-R for mutant S140A, or primer pair pEGFP-wtVP4-F/K180A-R and K180A-F/pEGFP-wtVP4-R for mutant K180A (Supplementary Table S1). Then fusion PCR was performed using mixed two overlapping fragments as template and primer pair pEGFP-wtVP4-F/pEGFP-wtVP4-R (Supplementary Table S1). The desired VP4 mutant fragments were subcloned into pEGFP-C2 vector. To generate multiple mutants for each amino acid of C-terminal peptide ^239^HLAMA^243^ of VP4, a serial of primers with MNN degenerate codon (where N is A/C/G/T, M is A/C) were synthesized (Supplementary Table S1) and used as a reverse primer to amplify the VP4 with randomly mutated amino acids for each position. The amplified VP4 mutant fragments with NNK degenerate codon (where N is A/C/G/T, K is T/G) were subcloned into pEGFP-C2 vector using EcoRI/KpnI restriction sites, and then multiple random colonies were individually sequenced to obtain multiple mutations for each amino-acid position.

### TUNEL assay

DNA fragmentation was examined by TUNEL assay using an *In Situ* Cell Death Detection Kit (TMR red, Roche Applied Science, Indianapolis, IN) following the instruction with minor modifications. Briefly, DF-1 cells were infected with IBDV or transfected with recombinant plasmids pEGFP-wtVP4 or pEGFP-VP4-CΔ2. At 48 hpt, cells were fixed with 4% formaldehyde in PBS at room temperature for 10 min, permeated with 0.1% TX-100 for 2 min, rinsed twice with PBS, and then incubated with TUNEL reaction solution at 37 °C for 60 min. Cells were counterstained with DAPI and observed under the laser scanning confocal microscope.

### Immunogold labeling and transmission electron microscopy

IBDV-infected DF-1 cells were harvested by scraping and centrifuging at 1,000 × *g* for 10 min. The pellets were then embedded in 2% agarose gel and fixed with 4% formaldehyde and 0.5% glutaraldehyde in 0.1 M PBS for 2 h at 4 °C. The embedded cells were dehydrated, penetrated and embedded in Lowicryl K4M (Lowi, Waldkraiburg, Germany). Ultrathin sections of K4M-embedded cells were stained with anti-VP4 mAb followed by goat anti-mouse 10 nm colloidal gold conjugate. The immune-stained sections were counterstained with 2% uranium acetate for 5 min and 1% lead citrate for 5 min. Ultrastructure of gold-stained samples was observed by H-7650 transmission electron microscopy (Hitachi, Tokyo, Japan).

## Additional Information

**How to cite this article**: Zheng, X. *et al.* The C-terminal amyloidogenic peptide contributes to self-assembly of *Avibirnavirus* viral protease. *Sci. Rep.* **5**, 14794; doi: 10.1038/srep14794 (2015).

## Supplementary Material

Supplementary Information

Supplementary Movie S1

Supplementary Movie S2
