# Supplementary material for: The C-terminal amyloidogenic peptide contributes to self-assembly of Avibirnavirus viral protease
Source: Sci Rep. 2015 Oct 6;5:14794. doi: 10.1038/srep14794 (PMC4594098; doi:10.1038/srep14794)
Supplement: Supplementary Information [file srep14794-s1.pdf]

Supplementary Information for Scientific Reports

The C-terminal amyloidogenic peptide contributes to self-assembly of *Avibirnavirus* viral protease

Xiaojuan Zheng[1,2†], Lu Jia[1,2†], Boli Hu[3], Yanting Sun[1], Yina Zhang[1], Xiangxiang Gao[1], Tingjuan Deng[1], Shengjun Bao[1], Li Xu[1] and Jiyong Zhou[1,2,3*]

[1]Key Laboratory of Animal Virology of Ministry of Agriculture, Zhejiang University, Hangzhou 310058, PR China

[2]State Key Laboratory and Collaborative Innovation Center for Diagnosis and Treatment of Infectious Diseases, First Affiliated Hospital, Zhejiang University, Hangzhou 310003, PR China

[3]College of Veterinary Medicine, Nanjing Agricultural University, Nanjing 210095, PR China

[†] These authors contributed equally to this work

[*] Corresponding author: Tel: 86-571-8898-2698; Fax: 86-571-8898-2218; E-mail address: jyzhou@zju.edu.cn

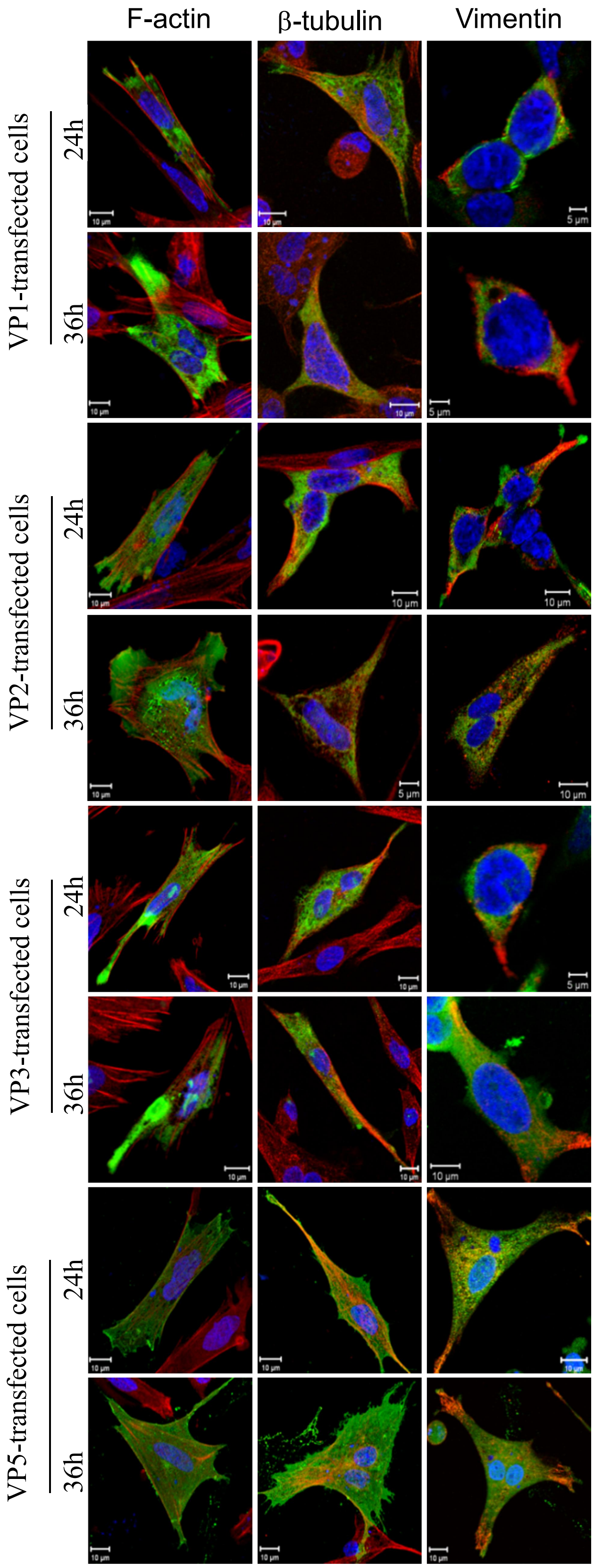

Supplementary Fig. S1 Subcellular relationships between other IBDV-encoded viral proteins and host cytoskeletal proteins. DF-1 cells transfected with pCI-VP1, VP2, VP3

and VP5 were applied for dual-staining IFA at 24 h and 36 h post transfection. Viral proteins were detected using mouse anti-VP1, VP2, VP3 or VP5 mAb followed by FITC-conjugated anti-mouse antibody. Host F-actin was probed by TRITC-phalloidin, host microtubule and intermediate filament were respectively detected by rabbit anti-β-tubulin and anti-vimentin antibodies followed by TRITC-conjugated goat anti-rabbit IgG. Nuclear DNA was stained with DAPI. The triple-stained cells were observed under a confocal microscope.

Supplementary Table S1 Primers for recombinant plasmid construction

Primer names	Sequence（5’→3’）	Positions[3]	Restriction sites
pEGFP-wtVP4-F[1]	AGAATTCGCCGACAAGGGGTA	1-14	*Eco*R I
pEGFP-wtVP4-R[2]	AAGGTACCctaAGCCATGGCAAGGT	716-729	*Kpn* I
pEGFP-VP4-C_{78-243}-F	GCGAATTCCTCCAACCTCCTTCT	232-246	*Eco*R I
pEGFP-VP4-N_{1-77}-R	TAGGTACCGTCTTCTCGCACGCCT	216-231	*Kpn* I
pEGFP-VP4-$C_{181-243}$-F	GAGAATTCCTCGCCACTGCACA	541-554	*Eco*R I
pEGFP-VP4-N_{1-180}-R	CCGGTACCCTTGGTGCTTCTA	528-540	*Kpn* I
pEGFP-VP4-CΔ1-R	AAGGTACCctaCATGGCAAGGTGGT	713-726	*Kpn* I
pEGFP-VP4-CΔ1′-R	AAGGTACCctaAGCGGCAAGGTGGT	713-726	*Kpn* I
pEGFP-VP4-CΔ2-R	AAGGTACCctaGGCAAGGTGGTA	712-723	*Kpn* I
PEGFP-VP4-CΔ3-R	CAGGTACCctaAAGGTGGTACTGGC	707-720	*Kpn* I
pCI-wtVP4-F	AGCTAGCatgGCCGACAAGGGGTA	1-14	*Nhe* I
pCI-wtVP4-R	CGGAATTCctaAGCCATGGCAAGGT	716-729	*Eco*R I
pCI-VP4-CΔ1-R	GAGAATTCctaCATGGCAAGGTGGT	713-726	*Eco*R I
pCI-VP4-CΔ1′-R	GAGAATTCctaAGCGGCAAGGTGGT	713-726	*Eco*R I
pCI-VP4-CΔ2-R	GCGAATTCctaGGCAAGGTGGTA	712-723	*Eco*R I
pCI-VP4-CΔ3-R	GCGAATTCctaAAGGTGGTACTGG	708-720	*Eco*R I
A5	TTAGCTAGCGGATACGATCGGTCTGACCCC	1-21	*Nhe* I
A3	ATTTGCGGCCGCGGGGACCCGCGAACGGATC	3241-3259	*Not* I
A-ΔMA-5	CAGTACCACCTTGCCGCATCAGAATTCAAA	2375-2410	-[4]
A-ΔMA-3	TTTGAATTCTGATGCGGCAAGGTGGTACTG	2375-2410	-
TNT-A5m	*CTATAGG*TGGTCAGTAGAGATCAGA	98-115	-
TNT-AT7	TAACTAGT*TAATACGACTCACTATAGG*TGGTCAGTA	98-106	*Spe* I
TNT-A3m	CCACGCGTGGGGACCCGCGAACGGAT	3242-3259	*Mlu* I
S140A-F	CTCCTATTGTGGGAAACGCTGGAAATCT	401-428	-
S140A-R	GGCTAGATTTCCAGCGTTTCCCACA	408-432	-
K180A-F	GCTTTAGAAGCACCGCACTCGCCACT	524-549	-
K180A-R	CAGTGGCGAGTGCGGTGCTTCTA	528-550	-
VP4-H239NNK[5]	CAGGTACCctaAGCCATGGCCAGMNNGTA	722-729	*Kpn* I
VP4-L240NNK	CAGGTACCctaAGCCATGGCMNNGTGGTA	722-729	*Kpn* I
VP4-A241NNK	CAGGTACCctaAGCCATMNNAAGGTGGTA	722-729	*Kpn* I
VP4-M242NNK	CAGGTACCctaAGCMNNGGCAAGGTGGTA	722-729	*Kpn* I
VP4-A243NNK	CAGGTACCctaMNNCATGGCAAGGTGGTA	722-729	*Kpn* I

Notes: The recognition sequences of restriction enzymes are underlined. The start and end codes are shown in box, and the sequences for introducing T7 promoter are shaded and italicized. 1 and 2: The F and R in the primer name indicate forward (F) and reverse (R) primer, respectively. 3: Positions of primers for VP4 amplification refer to the nucleotide regions of full-length VP4 (1-729nt), while positions of primers for segment A-associated recombinant plasmids refer to the nucleotide regions of full-length A segment (1-3259nt). 4: no restriction enzymes recognizing site. 5: MNN in the primers indicates degenerate codons for each amino acid position, where N=A/C/G/T, and M=C/A.

Video Legend

Supplementary Video S1. Self-assembly dynamics of EGFP-wtVP4 protein within the cytoplasm and nucleus. DF-1 cells were transfected with pEGFP-wtVP4 plasmid and subjected to live-cell imaging at 8 h following transfection. Intervals=1 min, totally 768 minutes (1 min × 768 frames). Time series: 24 frames/s.

Supplementary Video S2. Diffuse expression of EGFP-VP4-CΔ2 protein induces extensive cell death. DF-1 cells were transfected with pEGFP-VP4-CΔ2 plasmid and subjected to live-cell imaging at 8 hours following transfection. Intervals=1 min, totally 456 minutes (1 min × 456 frames). Time series: 24 frames/s.
